# Surface Modification of Electrospun Scaffolds for Endothelialization of Tissue-Engineered Vascular Grafts Using Human Cord Blood-Derived Endothelial Cells

**DOI:** 10.3390/jcm8020185

**Published:** 2019-02-04

**Authors:** Diana Catalina Ardila, Jr-Jiun Liou, David Maestas, Marvin J. Slepian, Michael Badowski, William R. Wagner, David Harris, Jonathan P. Vande Geest

**Affiliations:** 1 Department of Bioengineering, University of Pittsburgh, Pittsburgh, PA 15219, USA; dca13@pitt.edu (D.C.A.); jrl101@pitt.edu (J.-J.L.); wagnerwr@upmc.edu (W.W.); 2 Department of Biomedical Engineering, Johns Hopkins University, Baltimore, MD 21231, USA; dmaesta1@jhmi.edu; 3 Sarver Heart Center, The University of Arizona, Tucson, AZ 85721, USA; slepian@email.arizona.edu; 4 The Arizona Center for Accelerated BioMedical Innovation, University of Arizona, Tucson, AZ 85721, USA; 5 BIO5 Institute for Biocollaborative Research, The University of Arizona, Tucson, AZ 85721, USA; 6 Interventional Cardiology, University of Arizona, Tucson, AZ 85721, USA; 7 Arizona Health Science Center Biorepository, University of Arizona, Tucson, AZ 85724, USA; badowski@email.arizona.edu (M.B.); davidh@email.arizona.edu (D.H.); 8 McGowan Institute for Regenerative Medicine, University of Pittsburgh, Pittsburgh, PA 15219, USA; 9 Department of Surgery, University of Pittsburgh, Pittsburgh, PA 15219, USA; 10 Department of Immunobiology, Arizona Health Science Center Biorepository, University of Arizona, Tucson, AZ 85724, USA; 11 Vascular Medicine Institute, University of Pittsburgh, Pittsburgh, PA 15219, USA

**Keywords:** Vascular tissue engineering, umbilical cord blood, human cord blood-derived endothelial cells, endothelialization, vascular graft

## Abstract

Tissue engineering has gained attention as an alternative approach for developing small diameter tissue-engineered vascular grafts intended for bypass surgery, as an option to treat coronary heart disease. To promote the formation of a healthy endothelial cell monolayer in the lumen of the graft, polycaprolactone/gelatin/fibrinogen scaffolds were developed, and the surface was modified using thermoforming and coating with collagen IV and fibronectin. Human cord blood-derived endothelial cells (hCB-ECs) were seeded onto the scaffolds and the important characteristics of a healthy endothelial cell layer were evaluated under static conditions using human umbilical vein endothelial cells as a control. We found that polycaprolactone/gelatin/fibrinogen scaffolds that were thermoformed and coated are the most suitable for endothelial cell growth. hCB-ECs can proliferate, produce endothelial nitric oxide synthase, respond to interleukin 1 beta, and reduce platelet deposition.

## 1. Introduction

Coronary heart disease (CHD) resulting from atherosclerosis remains the leading cause of death in the United States [1]. CHD alone caused approximately 1 of 7 deaths in 2013, and 660,000 hospitalizations as a result of myocardial infarctions [2]. Once a coronary artery is compromised, a vascular bypass is an option to restore blood flow to tissues distal of the restriction or blockage [3]. Artery bypass graft surgery procedures usually involve the replacement of a coronary artery with an autologous vessel such as the saphenous vein or internal mammary artery [4,5]. Nevertheless, autologous grafts are not always available due to preexisting vascular conditions, or their use in a prior bypass operation [6]. The commercial alternatives to autologous grafts are vessels made of synthetic materials such as polytetrafluoroethylene (PTFE) or polyethylene terephthalate (known as Dacron). These grafts have been implemented with some success in medium and large diameter vessel replacement, but their efficacy is limited when used in small diameter vessels (<6 mm) where low blood flow makes the synthetic graft difficult to cellularize and more prone to thrombus formation, calcification, and intimal hyperplasia [3,6,7]. Therefore, there is an urgent need for small diameter graft alternatives that are able to support cell growth and match the mechanical properties of a native coronary artery while reducing the risk of acute thrombus formation and restenosis [8,9].

Recently, tissue engineering has gained attention as an alternative approach for developing small diameter vascular grafts from biocompatible natural or synthetic polymers [10,11]. Different methods have been used to fabricate tissue-engineered vascular grafts (TEVGs), such as solvent casting, phase separation, and electrospinning [10]. Electrospinning, in particular, has been extensively utilized to create fibrous scaffolds produced from nonwoven meshes containing fibers with diameters from micrometers to nanometers [10,12,13,14]. Its ability to combine the mechanical durability of synthetic materials with the cell compatibility of natural polymers makes electrospinning particularly attractive for TEVGs [10]. Some studies have reported the application of polycaprolactone scaffolds for the fabrication of TEVGs [15,16,17]. Previously we found that the incorporation of both gelatin and fibrinogen enhances the compliance which is critical for a functional vascular graft [18]. To further enhance the biocompatibility of a scaffold, surface modification has been shown to promote cell attachment, viability, and biological response of cells in the biomaterial. One of the common surface modification techniques is extracellular matrix protein coating [11].

For a TEVG to be successful, it needs to promote the formation of a healthy endothelial cell monolayer in the lumen of the graft [19]. Endothelial cells play a critical role in the control of vascular function. They participate in all aspects of vascular homeostasis, but they are also critical in physiological or pathological processes like regulation of vascular tone, inflammation, and in the prevention of thrombosis and intimal hyperplasia [20,21,22]. Therefore, an establishment of a healthy endothelium is crucial for the long-term success of a TEVG [19]. Endothelial progenitor cells as a source of cells for TEVG may be obtained from many sources including bone marrow, peripheral blood, and cord blood. Cord blood is an ideal source, since it has not been exposed to exogenous conditions that could diminish its numbers or function, such as diabetes, autoimmune disease, or old age [23]. In recent years, human umbilical cord blood-derived endothelial cells (hCB-ECs) have been increasingly used for tissue engineering applications, due to the highly proliferative capacity of these stem and differentiated cells in vitro [24]. An increased rate of proliferation results in less time and costs involved when “seeding” the grafts. A variety of studies have shown that hCB-ECs develop into a homogeneous population of endothelial cells that can be passaged 50–100 times before reaching senescence or losing the differentiated endothelial cell phenotype [24,25,26] as compared to other sources of endothelial precursor cells (such as adult peripheral blood), owing in part to the “young” age of the CB cells, which again decreases overall cost. In addition, a younger source of stem cells and EC derived from those stem cells is advantageous with respect to potential negative epigenetic effects observed in adult sources of cells. Studies comparing the protein profile of hCB-ECs with human umbilical vein endothelial cells (HUVECs) concluded that hCB-ECs display a higher proliferative capacity, higher sensitivity to angiogenic factors, and a differentiated production of the antioxidant enzyme manganese superoxide dismutase, which makes hCB-ECs more tolerant to oxidative stress than HUVECs [24,27,28,29,30,31,32] and more likely to survive in vivo.

In this study, gelatin/fibrinogen/polycaprolactone scaffolds were electrospun and surface-modified through a thermoforming process and coated with a blend of collagen IV and fibronectin. hCB-ECs were tested for their ability to proliferate and remain on the surface of the scaffold in formation of a monolayer. We also assessed whether the attached cells could function similar to a mature endothelial cell layer quantifying their response to pro-inflammatory cytokine interleukin 1 beta (IL-1β), the production of endothelial nitric oxide synthase (eNOS), and antithrombotic capacities as compared to HUVECs. This study evaluates the suitability of a surface modified biomaterial for endothelialization of a vascular graft as well as the use of hCB-ECs as a cell source for cardiovascular tissue engineering applications.

## 2. Materials and Methods

### 2.1. Cell Isolation and Cell Characterization

Human umbilical vein endothelial cells (HUVECs) (ATCC, Manassas, VA, USA) were purchased and cultured per manufacturer’s instructions as a positive control. Human cord blood-derived endothelial cells (hCB-ECs) were isolated as follows and de-identified umbilical cord blood (*n* = 3 donors) was obtained from the University of Arizona Biorepository per protocols approved by the University of Arizona’s Institutional Review Board (IRB). After collection, the isolation and differentiation procedures followed those described by Javed et al. (2008) [33], with minor modifications. Briefly, cord blood (20–100 mL) was diluted 1:1 with Hank’s balanced salt solution (HBSS), and then overlaid onto an equivalent volume of Histopaque 1077 (Sigma-Aldrich, St. Louis, MO, USA). To isolate mononuclear cells, the diluted cord blood was centrifuged for 30 min at room temperature at 740× *g*. The isolated mononuclear cells were washed and resuspended in 12 mL complete EGM-2 plus medium (Lonza, Basel, Switzerland), supplemented with 15% fetal bovine serum (Thermo Fisher Scientific, Pittsburgh, PA, USA). The cells were seeded onto 3 wells of a 6-well plate precoated with rat tail collagen type I (Life Technologies, Carlsbad, CA, USA) and maintained in a humidified environment at 37 °C and 5% CO_2_. The medium was changed daily for the first 7 days and every other day until the first passage. Colonies of endothelial cells appeared between 5 and 22 days of culture.

Cell identity was confirmed by flow cytometry and immunocytochemistry (ICC) as previously described [31] using mouse anti-human primary antibodies (BD Biosciences, Franklin Lakes, NJ, USA). For flow cytometry, fluorescein isothiocyanate (FITC)-conjugated CD31 antibodies, allophycocyanin (APC)-conjugated CD105 antibodies, and phycoerythrin (PE)-conjugated CD45 antibodies were used. Fluorescence intensity per cell produced by the bound antibodies was measured and cells were counted using the LSRII flow cytometer (BD Biosciences, Franklin Lakes, NJ, USA). For each flow cytometry experiment, 10,000–30,000 cells were gated from a population of 500,000 cells. For ICC, cells were cultured on glass coverslips coated with rat tail collagen I and the immunostaining was performed using the FITC conjugated CD31 antibodies. Cell nuclei were counterstained using VECTSHIELD^®^ 4′,6-diamidino-2-phenylindole (DAPI)-containing mounting media (Vector Laboratories, Burlingame, CA, USA). For all experiments performed, cells from passage 2–6 were used. Detailed information on the antibodies used is provided in Table S1.

### 2.2. Scaffold Fabrication

Flat sheet scaffolds were fabricated by electrospinning. Briefly, polycaprolactone, (PCL, 80,000 MW; Sigma-Aldrich, St. Louis, MO, USA), gelatin extracted from porcine skin (Sigma-Aldrich, St. Louis, MO, USA), and fraction I bovine fibrinogen (Sigma-Aldrich, St. Louis, MO, USA) were mixed at a ratio of 50% PCL:40% gelatin:10% fibrinogen w/w (hereafter PCL-GF) [18,34,35]. The polymeric blend was dissolved in 1,1,1,3,3,3-Hexafluoro-2-propanol (Sigma-Aldrich, St. Louis, MO, USA) to create a 10% w/v solution under constant stirring until completely homogeneous. The solution was loaded into a 5 mL BD syringe with a 23-gauge stainless steel dispensing blunt tip needle attached (CML Supply, Lexington, KY, USA). The syringe was then loaded into a NE-1000 single syringe pump (New Era Pump Systems Inc, Farmingdale, NY, USA) set to a pump rate of 30 μL/min. The distance from the needle tip to the target was 8 cm. The polymeric solution was electrospun with an applied voltage of 15 kV, onto glass coverslips attached to a metallic target to create fine fibers. The resulting flat sheets were cross-linked in 25% glutaraldehyde (Sigma-Aldrich, St. Louis, MO, USA) in vapor phase for 24 h. The glutaraldehyde was evaporated in a convection oven overnight at 42 °C, which is a temperature below the denaturation point of gelatin and fibrinogen [36,37]. Additionally, the scaffolds were rinsed with deionized water to remove any cross-linker residues and uncross-linked gelatin [35]. This method of removing glutaraldehyde was previously demonstrated no cytotoxicity of the cross-linked scaffolds for the proliferation of smooth muscle cells [35].

### 2.3. Surface Modification

After cross-linking, the scaffolds were thermoformed by a technique previously reported by our group [38]. The scaffolds were immersed in a water bath at 45 °C for 2 min, and then quickly placed in between two glass slides. A pressure of approximately 45 mmHg was applied using a 25 mm wide binder clip while the scaffolds were immersed once more in the 45 °C water bath for 5 min [39]. The scaffolds were equilibrated at room temperature for 10 min before the pressure was released.

Thermoformed scaffolds were sterilized with 70% ethanol for 1 h, rinsed with sterile 1× PBS and then placed under UV light (254 nm) for 1 h. The sterile scaffolds were coated with a solution 1:1 of collagen IV (Sigma-Aldrich, St. Louis, MO, USA) and fibronectin (Sigma-Aldrich, St. Louis, MO, USA) in HBSS with a final concentration of 5 µg/mL for 24 h at 4 °C. This coating blend has previously shown to promote hCB-EC growth when compared to Collagen IV alone (Figure S1). The coating solution was carefully rinsed with sterile 1× PBS. The scaffolds were then designated as thermoformed and coated (hereafter TC). The combined surface modification with thermoforming and coating was selected because our preliminary data showed that scaffolds thermoformed and coated are most favorable for cell attachment and spreading when compared to thermoformed alone or coated alone (Figure S2).

### 2.4. Scaffold Imaging

PCL-GF scaffolds, both nontreated (hereafter NT) and thermoformed, were imaged using atomic force microscopy (AFM). All AFM data was collected with a Cypher (Asylum Research and Oxford Instruments company, Santa Barbara, CA, USA) using AC Topography mode in air and NSC15 tapping mode probes (Mikromasch, Watsonville, CA, USA). For each scaffold treatment, 3 samples were imaged in 4 random areas of 25 µm^2^ along the mesh of fibers, and roughness values (Ra) were calculated after applying a first order flatten on all surfaces using Asylum Research software version 14.13.134.

Scanning electron microscopy (SEM) was performed on both TC and NT scaffolds. Briefly, samples were mounted onto aluminum stubs, grounded with silver paint, and sputter-coated with 6 nm gold/palladium (Cressington Sputter-coater Auto 108, Cressington, Watford, UK). En face views of the samples (*n* = 6 scaffolds each) were imaged using a JEOL JSM-6335F scanning electron microscope (JEOL USA, Peabody, MA, USA) at 3 kV at a magnification of 10,000×. The SEM images were binarized and the porosity was calculated as the ratio of the total number of fiber pixels to the total number of pixels in the image. The fiber diameter was calculated by manually measuring the diameter of 120 randomly selected fibers per scaffold treatment via freehand lines superimposed over the SEM images in ImageJ.

Multiphoton microscopy was used for the three-dimensional imaging of our electrospun scaffolds. The Pitt Advanced Intravital Microscope (AIM) for multiphoton imaging at the University of Pittsburgh Soft Tissue Biomechanics Laboratory allowed us to measure the change in scaffold thickness. This Olympus BX51 upright laser scanning microscope (Olympus, Tokyo, Japan) was coupled to a 120-fs tunable pulsed Titanium-Sapphire laser (Coherent Inc, Santa Clara, CA, USA) and an Olympus XLUMPLFL 20× water immersion objective with a numerical aperture of 0.9 [40,41]. The fibers were imaged centering the laser at 780 nm to excite the autofluorescence signal from the scaffolds (NADH), split with a 568 nm dichroic mirror, and collected through a 525/50 nm bandpass filter. The signal was collected over a 400 μm × 400 μm field of view at 2-μm z-step-size along the scaffold thickness.

### 2.5. Effect of Surface Modification on Cell Growth

hCB-ECs and HUVECs were seeded in NT and TC scaffolds at 10,000 cells/scaffold and cultured for 7 days. The culture medium was changed every other day and cultures were maintained in a humidified environment at 37 °C and 5% CO_2_. Cell growth was evaluated after 7 days of culture. A sample of approximately 25 mm^2^ was cut from each scaffold, and cell number was measured by MTS assay. Briefly, cell-seeded scaffolds were incubated in culture medium supplemented with CellTiter 96 AQueous One Solution Cell Proliferation Assay (Promega, Madison, WI, USA) at 37 °C for 4 h. Supernatant was collected and the absorbance at 490 nm was recorded. Background absorbance from the NT and TC scaffolds was obtained from nonseeded scaffolds. Cell number was calculated based on our calibration curves (Figure S3).

For cell imaging, the scaffolds were fixed with 2% formaldehyde and stained with Alexa Fluor^®^ 568 phalloidin (Life Technologies, Carlsbad, CA, USA) to visualize f-actin following the manufacturer’s instructions. To stain the nuclei, the scaffolds were treated for 24 h with VECTASHIELD^®^ DAPI mounting medium (Vector Laboratories, Burlingame, CA, USA). The Pitt AIM with a 20× water immersion objective was used to visualize the cells growing in the scaffolds along the scaffold depth. The nuclei (blue), fibers (green), and f-actin (red) were imaged simultaneously and colocalized using three different photomultiplier tubes (PMTs). The laser was centered at λ = 780 nm to excite simultaneously DAPI, the autofluorescence signal from the scaffolds (NADH), and Alexa Fluor^®^ 568. In the first PMT, the DAPI signal was split with a 505 nm dichroic mirror and collected through a 460/80 bandpass filter. In the second PMT, the signal from the scaffolds was split with a 568 nm dichroic mirror and collected through a 525/50 bandpass filter. Alexa Fluor^®^ 568 signal was acquired in the third PMT by splitting the signal with a 568 nm dichroic mirror and collecting using a 607/70 bandpass filter. The colocalized image stacks from the cell nuclei, the fibers, and f-actin were merged to visualize the cell location in the scaffolds. Maximum intensity projections (MIPs) were produced to visualize the total number of cells in the field of view. The percentage of cell infiltration was calculated as the ratio of the length that cells migrating through the flat sheet from the top to the bottom relative to the flat sheet thickness. For instance, in a cell-seeded flat sheet, we obtained 20 images every 2 µm in the Z-direction; the flat sheet had an estimated thickness of 40 µm. If the cells only appear in the first five images, it means the cells migrated through the first 2 × 5 = 10 µm. Therefore, the percentage of cell infiltration is calculated as 10/40 = 25% of that specific sample.

### 2.6. Platelet Activation and Platelet Adherence

De-identified human whole blood was obtained at the University of Arizona Sarver Heart Center in accordance with IRB-approved protocols. Briefly, 30 mL of whole blood was drawn through venipuncture into 3 mL of acid citrate dextrose A. Subsequently, the blood was centrifuged at 500× *g* for 15 min to obtain platelet-rich plasma, which was gel-filtered through a column of Sepharose 2B beads (GE Healthcare Life Sciences, Marlborough, MA, USA) with HEPES-modified Tyrode’s buffer (Boston BioProducts Inc, Ashland, MA, USA), to collect erythrocyte-free platelets. With the aim of quantifying the activation of platelets caused by the contact with cell-seeded scaffolds, the platelet concentration was diluted to 20,000 platelets/μL in HEPES-modified Tyrode’s buffer, with 3 mM CaCl_2_ added 10 min prior to any experiment to avoid platelet-platelet activation.

Platelet activity state (PAS) was measured using a modified-prothrombinase assay reported in the literature [42,43]. Briefly, after 7 days of hCB-EC and HUVEC culture in NT or TC scaffolds, a sample of 20,000 platelets/μL was perfused onto the surface of the cell-seeded scaffolds and incubated at 37 °C for 0, 1, and 2 h. After incubation, 100 pM factor Xa, 5 mM calcium, and 200 nM acetylated prothrombin, were added and incubated for 10 min. Thrombin generation was quantified through spectrophotometric analysis over 7 min at an absorbance wavelength of 405 nm to obtain the PAS values, using Chromozym-TH (Roche, Penzberg, Germany) as the thrombin-specific chromogenic peptide substrate. The platelets in a control tube were fully activated by sonication. Each PAS value was normalized to the PAS value obtained in the control tube [43].

Samples were prepared for scanning electron microscopy (SEM) following Merkle et al., with minor modifications [43]. Nonadherent platelets were gently washed away with 1× DPBS, and the samples were immediately fixed with a solution composed of 2% formaldehyde and 2% glutaraldehyde in 1× DPBS for 4 h. The samples were washed with 25% ethanol for 15 min and dehydrated in 50%, 75%, and 100% ethanol at 15-min intervals. The samples were treated with critical point drying in CO_2_ using an E3100 Critical Point Dryer (Quorum Technologies LTDA, England). Each sample was then mounted with carbon tape and coated with platinum for 30 s in a Hummer 6.2 argon gas sputter system (Anatech LTD, Battle Creek, MI, USA). All samples were imaged using a field emission SEM (Hitachi, Tokyo, Japan) with the working distance and accelerating voltage set to enhance the contrast of adhered platelets against the cell surfaces. Platelet count on scaffolds was determined using ImageJ. For each manual count, 10 random 40,000 µm^2^ sections of area were selected and averaged to estimate the relative adherence of platelets on each scaffold treatment of each cell type [43,44]. Platelets were characterized based on their characteristic size (2.3–4.3 μm) and shape [45]. Aggregated platelets were excluded during platelet counting. Percentage of cell coverage was quantified by segmenting the SEM images and dividing the pixels corresponding to the cell area by the total number of pixels in the image.

### 2.7. Response to Interleukin 1 Beta (IL-1β) and Endothelial Nitric Oxide Synthase (eNOS) Production

To examine the response to IL-1β, after 4 days of hCB-EC and HUVEC culture in NT or TC scaffolds, recombinant human IL-1β (Life Technologies, Carlsbad, CA, USA) was added to each scaffold at a final concentration of 0.5 ng/mL. After 72-h treatment, the scaffolds with or without IL-1β were fixed with 2% formaldehyde. To examine the eNOS production, a separate set of cultures of hCB-EC and HUVEC in NT or TC scaffolds were fixed with 2% formaldehyde at day 7.

An in-cell enzyme-linked immunosorbent assay (ELISA) was performed using mouse anti-intercellular adhesion molecule 1 (ICAM-1; Abcam, Cambridge, United Kingdom), mouse anti-vascular cell adhesion molecule 1 (VCAM-1; Abcam), and mouse anti-eNOS primary antibodies (Abcam). Primary antibodies were conjugated with HRP rabbit anti-mouse polyclonal secondary antibodies (Abcam) and one-step ultra TMB ELISA (Thermo Fisher Scientific, Waltham, MA, USA) was used to detect HRP activity following the manufacturer’s instructions. Absorbance was recorded at 450 nm in a Synergy H1 plate reader (BioTek, Winooski, VT, USA). Background absorbance from the NT and TC scaffolds was obtained from non-seeded scaffolds. The ICAM-1, VCAM-1, and eNOS results were normalized to the average cell number calculated from the MTS calibration curves (Figure S3). Detailed information on the antibodies used is provided in Table S1.

### 2.8. Statistical Analysis

All values are presented as the mean ± standard deviation unless otherwise specified. The statistical analysis was performed using the software package SPSS (IBM, Armonk, NY, USA). For surface roughness, fiber diameter, scaffold thickness, scaffold porosity, cell proliferation, and ELISA analyses, the statistical analysis was completed using two-way ANOVA with Tukey’s range tests to compare between two cell types and two scaffolds. For nitric oxide synthase production, platelet activity assay, and platelet adherence analyses, a one-way ANOVA was performed with a post hoc analysis using individual two-sample two-tailed *t*-tests. The significant difference was determined when the *p*-value < 0.05.

## 3. Results

### 3.1. Cell Characterization

**Figure 1:**
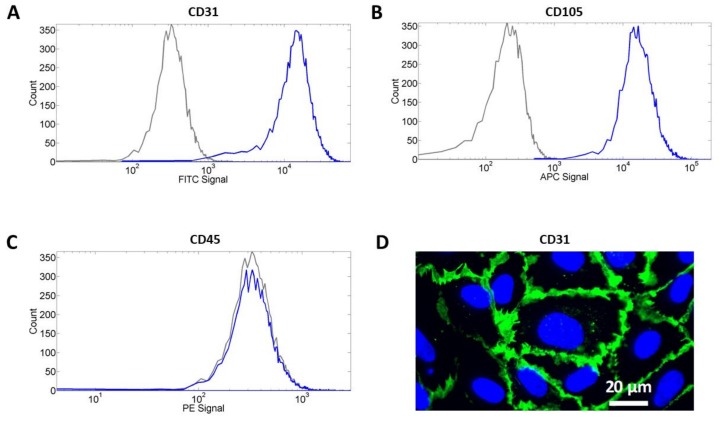
Characterization of human cord blood-derived endothelial cells (hCB-EC) monolayers using flow cytometry and immunocytochemistry. (**A**–**C**) Flow cytometry results show that hCB-ECs are positive for endothelial cell markers CD31 and CD105 while negative for CD45, a hematopoietic marker. (**D**) Immunocytochemistry of CD31 shows that the expression of CD31 is detected on the cell membrane (green—CD31; blue—nuclei). Scale bar = 20 μm.

hCB-ECs were derived from the monocytes collected from cord blood. After differentiation, cells were characterized using flow cytometry and immunocytochemistry. Figure 1 shows the results of the flow cytometry represented in histograms. The blue histogram characterizes the signal obtained from the cells treated with the antibodies and the gray histogram represents the cells that were not. A shift in the histogram indicates a relative increase in the average cell fluorescence. Antibodies against CD31 (Figure 1A) and CD105 (Figure 1B) were attached to the cell, confirming the expression of these two endothelial cell-specific markers in our hCB-ECs. In contrast, the blue histogram for the cells treated with the antibodies against CD45 (Figure 1C) did not shift, which can be interpreted as a lack of CD45 expression in our hCB-ECs. These results confirm that hCB-ECs were successfully isolated and there is no contamination from the hematopoietic lineage. Additionally, the immunocytochemistry corroborated the expression of CD31 exclusively in the cell membrane at the cell–cell junctions (Figure 1D).

### 3.2. Scaffold Characterization

AFM was performed in order to study the effect of thermoforming on surface roughness, which represents the average change in height on the surface with respect to a reference point. In this case, the reference points are the glass coverslips which the individual samples were laid on prior to imaging. No significant difference in roughness was found between the thermoformed and the nontreated scaffolds (299.30 nm ± 27.53 nm vs. 331.53 nm ± 23.44 nm).

**Figure 2:**
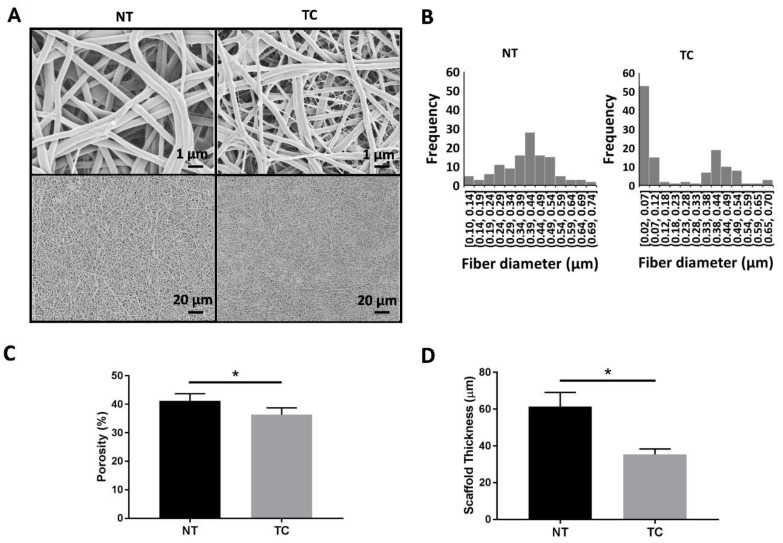
Effect of thermoforming and coating on polycaprolactone/gelatin/fibrinogen scaffolds. (**A**) Representative SEM images of nontreated (NT) or thermoformed and coated (TC) scaffolds. (**B**) Fiber diameter distribution in NT and TC scaffolds was calculated from the SEM images. NT scaffolds have a normal fiber size distribution with fibers ranging from 0.1 to 0.74 µm. The TC scaffolds have a bimodal fiber size distribution with thin fibers ranging from 0.02 to 0.12 µm, and thick fibers ranging from 0.12 to 0.7 µm (*n* = 6 scaffolds, 120 fibers). (**C**) Average porosity results calculated from the SEM images show a significant decrease in TC scaffolds (* *p* < 0.05; *n* = 6 scaffolds). (**D**) A significant reduction in thickness calculated from multiphoton images was found in the TC group (* *p* < 0.05; *n* = 6 scaffolds).

Figure 2 summarizes the effect of thermoforming and coating on scaffold microstructure. Figure 2A shows representative SEM images of the nontreated (NT) and thermoformed/coated (TC) scaffolds. The scaffold porosity and fiber diameter were calculated from these SEM images. The histogram of fiber diameter shows a distribution of thinner fibers in the TC scaffolds (Figure 2B), which can be observed in the TC SEM images. A significant reduction in porosity was found in the TC group (36.3% ± 2.39% vs. 41.1% ± 2.54%) (Figure 2C). No significant difference was detected between the thicker fibers of the TC scaffolds and the fibers from NT scaffolds (0.42 µm ± 0.1 µm vs. 0.40 µm ± 0.12 µm). Scaffold thickness was calculated using the Z stacks from the multiphoton images. A significant reduction in thickness was found in the TC group (35.33 µm ± 2.74 µm vs. 61.33 µm ± 7.08 µm) (Figure 2D).

### 3.3. Effect of Surface Modification on Cell Attachment and Cell Growth

**Figure 3:**
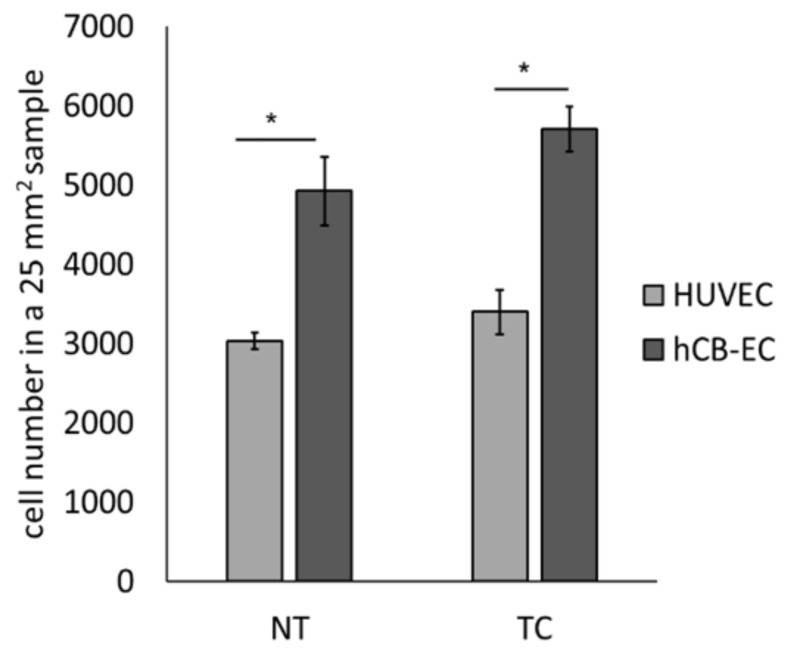
Cell number of hCB-ECs and human umbilical vein endothelial cells (HUVECs) cultured in NT or TC scaffolds at day 7. The cell number was calculated from the MTS calibration curves. A significant increase in hCB-ECs was found in NT and TC scaffolds when compared to HUVECs (* *p* < 0.05, *n* = 3).

To evaluate the effect of thermoforming and coating on endothelial cell growth, cell number of hCB-ECs and HUVECs in NT or TC scaffolds was evaluated at day 7 using MTS assays (*n* = 3 in each group). A significant increase in cell number was observed for hCB-ECs compared to HUVECs in NT (4.9 × 10^3^ ± 432.04 vs. 3.04 × 10^3^ ± 102.6, *p* = 0.003) and TC scaffolds (5.71 × 10^3^ ± 286.7 vs. 3.40 × 10^3^ ± 102.06, *p* = 0.001) (Figure 3).

**Figure 4:**
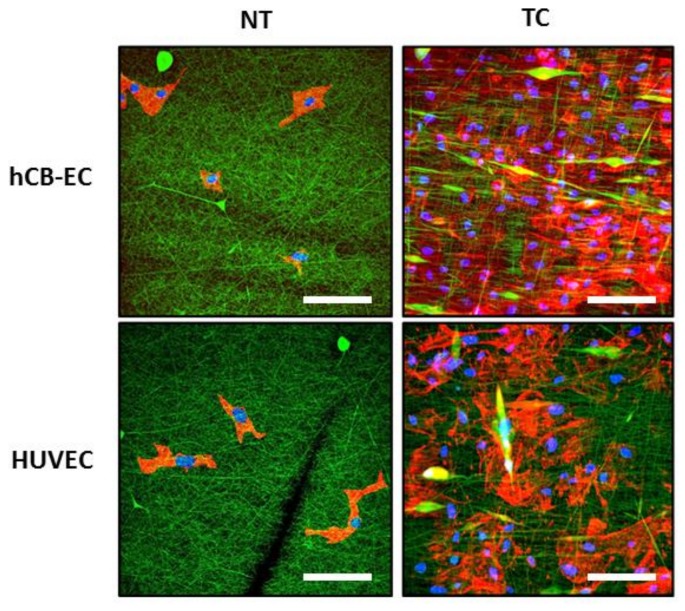
Maximum intensity projection multiphoton images of hCB-ECs or HUVECs cultured in nontreated (NT) or thermoformed/coated (TC) scaffolds after 7 days of culture (green—scaffolds; blue—nuclei; red—F-actin). The combined surface modification of thermoforming and coating favor cell spreading and cell attachment, especially for hCB-ECs. Scale bar = 100 μm.

To evaluate cell attachment, the cells in scaffolds were stained with DAPI (nuclei) and Phalloidin (F-actin) at day 7. Representative images of maximum intensity projections (MIPs) from each group is presented in Figure 4. We found that the combined surface modification of thermoforming and coating favor cell spreading and cell attachment compared to the NT scaffolds. When we calculated the percentage of cell infiltration from the multiphoton z-stack images, no significant difference was found between hCB-ECs cultured in TC and NT scaffolds (20.40% ± 4.5% vs. 17.2% ± 3.72%) or HUVECs cultured in TC and NT scaffolds (27.01% ± 4.34% vs. 26.01% ± 1.81%). In addition, no significant difference in infiltration was observed between hCB-ECs and HUVECs cultured in TC scaffolds (20.40% ± 4.5% vs. 27.01% ± 4.34%) or NT scaffolds (17.2% ± 3.72% vs. 26.01% ± 1.81%). Interestingly, we noticed that hCB-ECs have a lower cell infiltration than HUVECs in both NT and TC scaffolds.

### 3.4. Platelet Activation and Adhesion

**Figure 5:**
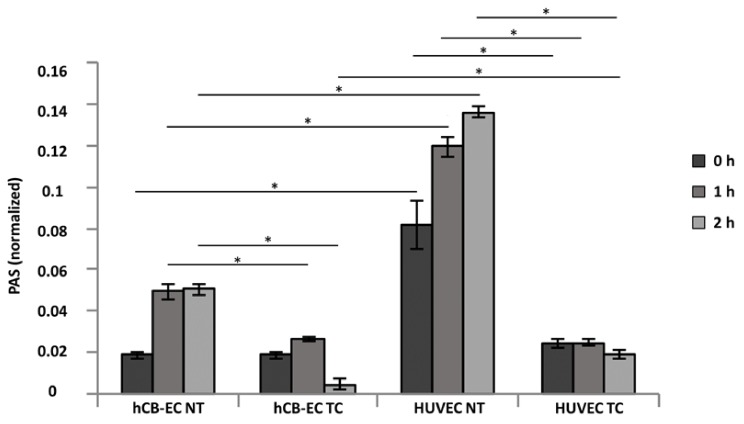
Platelet activity state (PAS) of hCB-EC or HUVEC seeded on thermoformed and coated (TC) scaffolds compared to nontreated (NT) scaffolds at 0, 1, and 2 h. TC groups have lower platelet activation when compared to NT groups; hCB-ECs have lower platelet activation than that of HUVECs (comparisons between hCB-EC and HUVEC cultured in same scaffold and comparisons between NT and TC of same cell type, * *p* < 0.05, *n* = 4).

The PAS was calculated for platelets on NT or TC scaffolds seeded with hCB-ECs or HUVECs using a prothrombinase assay. A sample of diluted platelets was pipetted on top of the seeded scaffolds and incubated for 0, 1, and 2 h. After incubation, factor Xa, Ca^2+^, and acetylated prothrombin were added to the samples and the generation of thrombin as a measurement of platelet activation was quantified by spectrophotometry. PAS values were defined as the ratio of thrombin generated by the samples to thrombin generated by sonication. In Figure 5, we found that in the TC samples, regardless of the cell type, PAS values decrease after 1h indicating a possible deactivation of platelets. On the contrary, in the NT scaffolds, the PAS values increased over time suggesting a constant activation of platelets. Comparisons were made between scaffolds within the same cell type and between cell types within the same scaffold. A significant difference in absorbance for thrombin generation was found for the NT and TC scaffolds seeded with hCB-ECs at 1 h (0.049 ± 0.003 vs. 0.026 ± 0.001, *p* = 0.0002) and 2 h (0.05 ± 0.003 vs. 0.0049 ± 0.002, *p* = 2.03 × 10^−10^) of platelet–prothrombinase incubation. Also, when comparing the thrombin absorbance results for the NT and TC scaffolds seeded with HUVECs, a significant difference in absorbance was found at 0 h (0.081 ± 0.01 vs. 0.024 ± 0.002, *p* = 0.001), 1 h (0.12 ± 0.04 vs. 0.024 ± 0.001, *p* = 9.83 × 10^−7^), and 2 h (0.135 ± 0.03 vs. 0.019 ± 0.001, *p* = 4.3 × 10^−10^). A significant decrease in thrombin generation was found in the NT scaffolds seeded with hCB-ECs compared to the ones with HUVECs at 0 h (0.018 ± 0.001 vs. 0.081 ± 0.01, *p* = 0.001), 1 h (0.049 ± 0.003 vs. 0.024 ± 0.001, *p* = 5.46 × 10^−7^), and 2 h (0.05 ± 0.003 vs. 0.135 ± 0.002, *p* = 2.44 × 10^−9^). For the TC scaffolds seeded with hCB-ECs, the thrombin generation by the perfused platelets was significantly lower when compared to the TC scaffolds seeded with HUVECs after 2 h of incubation (0.0049 ± 0.002 vs. 0.019 ± 0.001, *p* = 4.3 × 10^−10^).

**Figure 6:**
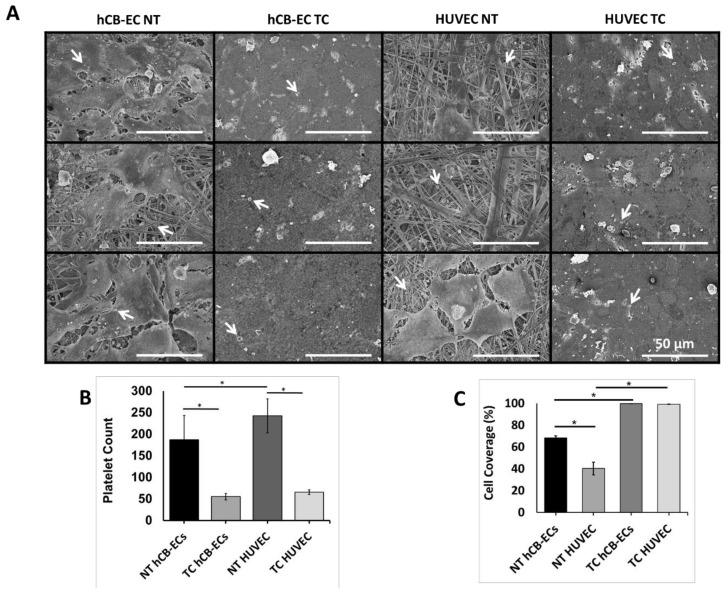
Assessment of platelet adhesion of hCB-ECs or HUVECs cultured on thermoformed/coated (TC) scaffolds compared to nontreated (NT) scaffolds. (**A**) Representative SEM images of each replicate in each experimental group. White arrows are pointing at platelets. Scale bar = 50 μm. (**B**) Platelet counts show that a significant reduction of platelet number in TC groups (* *p* < 0.05, *n* = 10). (**C**) Cell coverage results show that thermoforming and coating increase the cell coverage of scaffolds, and that hCB-ECs have higher cell coverage than HUVECs on NT scaffolds (* *p* < 0.05, *n* = 3).

Platelet adhesion is presented in Figure 6. Figure 6A shows representative SEM images taken for each replicate of NT and TC scaffolds seeded with either hCB-ECs or HUVECs. Cells seeded on NT scaffolds were not able to cover the entire surface, suggesting the presence of material fibers where many platelets are deposited. On the contrary, in TC scaffolds the cells were able to cover almost the entire surface of leaving almost no material fibers exposed reducing the adhesion of platelets. In NT scaffolds, HUVECs have fewer cells attached to the surface than hCB-ECs, leaving more fibers exposed. Similarly, in TC scaffolds HUVECs present more gaps in between the cells resulting in higher platelet adhesion. Figure 6B shows the results of the platelet counts from the SEM images. A significant reduction was found in the number of platelets adhered to both NT (186.9 ± 56.39 vs. 242.5 ± 39.22, *p* = 0.025) and TC (55.4 ± 7.31 vs. 65.77 ± 5.51, *p* = 0.0033) scaffolds when hCB-ECs were seeded. When assessing the differences between scaffold types, the TC had a significantly lower number of platelets deposited as compared to the nontreated with either hCB-ECs (55.4 ± 7.31 vs. 186.9 ± 56.39, *p* = 0.000017) or HUVECs (65.77 ± 5.51 vs. 242.5 ± 39.22, *p* = 8.4 × 10^−11^). Figure 6C presents the quantification of cell coverage calculated from the SEM images. A significant increase in cell coverage was found for hCB-ECs growing in TC compared to NT surfaces (99.73% ± 0.053% vs. 66.72% ± 2.07%, *p* = 0.00023). Similarly, HUVEC presented a significant increase in coverage of TC compared to NT surfaces (99.08% ± 0.13% vs. 40.19% ± 5.83%, *p* = 0.0003). hCB-ECs show significantly more covered area than HUVEC seeded in NT scaffolds (66.72% ± 2.07% vs. 40.19% ± 5.83%, *p* = 0.037).

### 3.5. Inflammatory Response and eNOS Production

**Figure 7:**
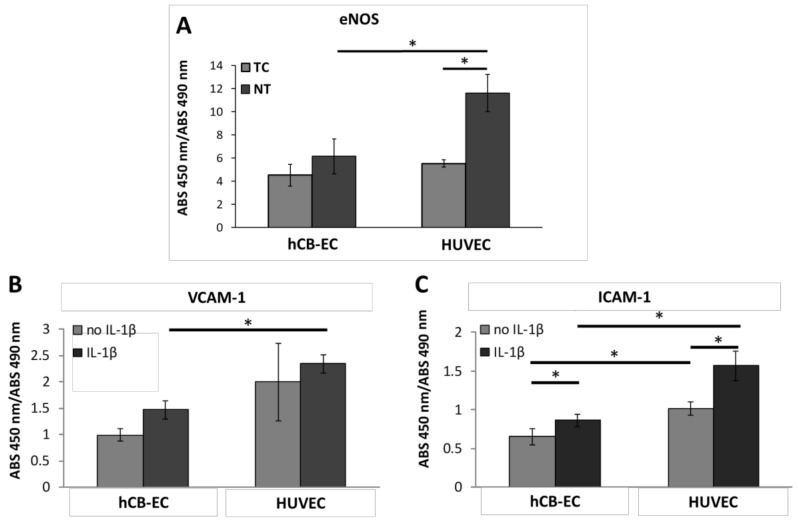
Endothelial nitric oxide synthase (eNOS) production and response to IL-1β of hCB-EC- and HUVEC-seeded scaffolds. (**A**) eNOS ELISA was quantified and normalized to MTS assay. The eNOS production is significantly lower in hCB-ECs as compared to HUVECs when seeded on NT scaffolds (* *p* < 0.05; *n* = 4). (**B**) Anti-vascular cell adhesion molecule 1 (VCAM-1) ELISA in TC scaffolds with or without the addition of 0.5 ng/mL of IL-1β show that HUVECs have higher response compared to hCB-ECs (* *p* < 0.05; *n* = 4). (**C**) ELISA of anti-intercellular adhesion molecule 1 (ICAM-1) shows that HUVECs have higher response compared to hCB-ECs (* *p* < 0.05; *n* = 4).

To determine if the cells growing on TC scaffolds can produce eNOS in static conditions, the expression of eNOS was quantified after 7 days in culture using in-cell ELISA. A baseline for eNOS expression was established using NT scaffolds. The absorbance at 450 nm as a result of eNOS production was normalized to the absorbance of MTS assay (490 nm) of hCB-ECs and HUVEC after 7 days growing in the TC and NT scaffolds. The eNOS production was found to be significantly lower in hCB-ECs as compared to HUVECs (6.14 ± 1.04 vs. 11.61 ± 1.04; *p* = 0.024) when the cells are seeded on NT scaffolds (Figure 7A). The production of eNOS by HUVECs growing in the TC scaffolds is significantly lower as compared to their eNOS production when these cells are seeded in NT surfaces (5.52 ± 0.48 vs. 11.61 ± 1.04; *p* = 0.006).

To study the inflammatory response of hCB-ECs and HUVEC on the TC scaffolds, IL-1β was added to the culture and the expression of ICAM-1 and VCAM-1 was quantified using in-cell ELISA. For the hCB-ECs and HUVECs seeded onto TC scaffolds, the addition of IL-1β increased the absorbance at 450 nm. An increase in absorbance is correlated with the expression of inflammation associated proteins VCAM-1 and ICAM-1. All absorbance values were normalized by the MTS assay (490 nm) of hCB-ECs and HUVEC after 7 days growing in the TC scaffolds. A significant decrease in VCAM-1 production was found comparing hCB-ECs to HUVECs after the addition of IL-1β (1.47 ± 0.17 vs. 2.34 ± 0.17; *p* = 0.00058) (Figure 7B). The expression of ICAM-1 is also significantly lower in hCB-EC as compared to HUVEC seeded scaffolds without (0.65 ± 0.11 vs. 1.01 ± 0.089; *p* = 0.0015) and with IL-1β stimulation (0.86 ± 0.07 vs. 1.56 ± 0.19; *p* = 0.00067) (Figure 7C).

## 4. Discussion

The purpose of this study was to evaluate the surface modification of a biomaterial to promote endothelialization of electrospun scaffolds by thermoforming and coating with collagen IV and fibronectin and to quantify the performance of cord blood endothelial cells on our biomaterial surface and its potential for vascular tissue engineering applications.

Our research team was able to isolate mononuclear cells from human umbilical cord blood, differentiate them into endothelial cells, and culture hCB-ECs on electrospun scaffolds while maintaining endothelial cell phenotype. Our findings suggest that scaffolds composed of polycaprolactone/gelatin/fibrinogen that have been surface modified by thermoforming and coating with a mixture 1:1 collagen IV to fibronectin will promote the formation of an endothelial monolayer.

In these scaffolds cell growth is encouraged, resulting in a monolayer of endothelial cells. hCB-ECs proliferate more robustly on our surface modified scaffolds than HUVECs, and the maintenance of the endothelial cell function of both hCB-ECs and HUVECs is comparable. hCB-EC-seeded scaffolds upregulated the adhesion molecules ICAM-1 and VCAM-1 when stimulated with the pro-inflammatory cytokine IL-1β. Our hCB-ECs also displayed the ability to produce the vasoregulatory- and hemocompatibility-related enzyme eNOS and reduce platelet adhesion and activation.

Our source of endothelial cells is cord blood, which is characterized by a unique richness in highly proliferative stem and progenitor cells [46]. Cord blood is readily available, can be collected noninvasively without risk to the mother or infant donor, and can be tested and preserved for long periods of time for future use [47]. This source of endothelial cells has many advantages compared to bone marrow and peripheral blood. Some of the main advantages are the proliferative capacity of the cord blood progenitor cells, higher number of stem cells per volume of blood, and a higher tolerance of cord blood progenitor cells of human leukocyte antigen mismatches [48]. Moreover, hCB-ECs can be used in the treatment of acute ischemic disease, in aging patients, and patients with risk of cardiovascular disease, as opposed to peripheral blood derived endothelial cells [48]. The endothelial cells from cord blood will be treated as a nonautologous source of cells that has to be donor matched [31,49]. Generally, it has been accepted that human leukocyte antigen mismatches are better tolerated with cells derived from cord blood as oppose to bone marrow, resulting in less graft versus host disease [48]. This suggests that endothelial cells derived from cord blood are more adaptable as they are young and not fully differentiated in early passages, therefore they are suitable for clinical applications.

Different efforts have been made for the endothelialization of small diameter vascular grafts, and many of them have used surface modified scaffolds fabricated with synthetic polymers which have excellent biomechanical properties but low biocompatibility [5,50,51,52,53,54,55,56,57,58]. Surface modifications can reduce hydrophobicity of the material and therefore increase biocompatibility. One of the most popular techniques for improving biocompatibility and endothelial cell adhesion is immobilization of peptide ligands onto the grafts. Peptide sequences such as RGD, GRGDSP, and DGEA have been utilized since they interact directly with endothelial cell receptors and increase cell attachment [59].

In this work, we demonstrated that a scaffold made of a blend of synthetic and natural polymers including polycaprolactone, gelatin, and fibrinogen following by thermoforming and coating with a mixture of collagen IV and fibronectin improves hCB-EC cell growth. This unique hybrid biomaterial possesses the RGD peptide from gelatin [60,61], fibrinogen [62], and fibronectin [63]; GRGDSP peptide from fibronectin [64]; DGEA peptide from gelatin [65]; and FYFDLR from collagen IV [66]. All these are important peptides recognized by integrins in the endothelial cell membrane [66,67,68,69]. Furthermore, the inclusion of polycaprolactone to the polymeric blend reduced fiber diameter which appears to promote cell attachment [70] and endothelial cell proliferation [71]. The thermoforming process aimed to smooth the surface of electrospun scaffolds. Surprisingly, thermoforming did not significantly alter the surface roughness in this study. However, the combined surface modification of thermoforming and coating decreased both scaffold porosity and scaffold thickness and altered the distribution of fiber diameters with the appearance of small fibers branching out from the major fibers. We observed better cell attachment in the thermoformed and coated scaffolds demonstrating that the surface-modified scaffolds favor the development of an endothelial cell monolayer. We noticed a difference between the cell number reported for the NT group in Figure 3 and that observed in Figure 4. We speculate this may be due to the staining and intensive washing required prior to multiphoton imaging but not required for an MTS assay, which may remove cells not strongly attached to the nontreated scaffolds. Further experiments should be run to confirm if the decreased strength of attachment of cells placed on NT materials is responsible for the lower cells present in Figure 4. Should this be the case, choosing the TC group would be advantageous given the increased levels of shear stress expected in vivo.

It is possible that the pressure applied to the scaffolds by thermoforming may lead to the production of a more compact fiber arrangement that aids with the retention of coating proteins and that the appearance of small fibers may be due to the coating process creating more contact points for the cells. Thus, the combined surface modification of thermoforming and coating facilitated better hCB-EC attachment and cell growth. Future work will be beneficial to understand the mechanisms by which this process is governed.

Our goal is not only to promote cell attachment and cell growth of hCB-ECs in our electrospun scaffolds but also to assess whether the attached cells can function as mature endothelial monolayers. For this purpose, we evaluated three important characteristics of a healthy endothelial cell layer under static conditions: the response to a pro-inflammatory stimulus, the production of eNOS (enzyme directly related with nitric oxide generation), and the antithrombotic capacity. We investigated the inflammatory response by exposing the hCB-EC- and HUVEC-seeded scaffolds to a pro-inflammatory cytokine IL-1β by assessing the production of VCAM-1 and ICAM-1. Our results show that in both cell types VCAM-1 and ICAM-1 were upregulated, demonstrating a positive response to IL-1β as occurring in the healthy vasculature [72,73,74,75]. Interestingly, hCB-ECs intrinsically have a lower production of the VCAM-1 and ICAM-1 which correlates to reduced recruitment of leucocytes/macrophages and thus a reduced probability of graft intimal hyperplasia and atherosclerosis in vivo [76,77].

In endothelial cells, eNOS is responsible for endothelium-derived nitric oxide (NO) production. In this study we measured eNOS production of hCB-ECs or HUVECs seeded on thermoformed/coated scaffolds and compared this with that of cells seeded on nontreated scaffolds. Both cell types expressed eNOS in static conditions in both the thermoformed/coated and nontreated scaffolds indicating that their endothelial cell phenotype is preserved. eNOS production in hCB-ECs is lower than the amount produced by HUVECs in both growing surfaces. These results are similar to those reported by Brown et al. (2009) who showed that hCB-ECs produced a significantly lower amount of eNOS than aortic ECs in static conditions [30]. Similarly, in the work of Yuan et al. (2016), the authors found that endothelial progenitor cells have a markedly lower expression of eNOS as well as activity levels compare to HUVECs and aortic ECs. They concluded that the expression of low levels of eNOS in endothelial progenitor cells compared to mature endothelial cells is due to their higher extracellular matrix deposition [78]. In our work, we also observed that eNOS is reduced significantly for HUVECs growing on TC scaffolds compared to NT scaffolds. This may due to the use of fibronectin in our coating causing the partial downregulation of eNOS in HUVECs which is in line with the study by Viji et al. (2009), who found that HUVECs growing on fibronectin-coated glass slides at a concentration of 50 µm/mL have a significantly reduced eNOS activity when compared to noncoated glass slides [79]. Yuan et al. also found that HUVEC growing in fibronectin-coated flasks have a significant reduction in eNOS mRNA and protein levels compared to HUVEC cultured on polystyrene flasks [78]. In vivo, the predominant physiological stimulus for eNOS active phosphorylation and subsequent NO segregation is wall shear stress [80,81,82]. It has been demonstrated that eNOS is produced in fewer quantities in endothelial cells cultured in static conditions [80,83,84]. In our work, no significant difference was found between the eNOS protein levels of hCB-ECs growing in NT and TC scaffolds, but it is possible that eNOS production may increase in hCB-EC-seeded scaffolds under flow conditions [30,85].

We also assessed antithrombotic capacity by evaluating platelet activation and deposition when in contact with either nontreated or thermoformed/coated scaffolds preseeded with hCB-ECs or HUVECs. Figure 5 shows the results of the platelet activity state (PAS) assay where the PAS values represent the amount of acetylated thrombin formed by the platelets in contact with the studied samples [43]. Overall, the PAS values over the course of 2 h are lower for the TC scaffolds showing a deactivation after the first hour. It is known that platelets are deactivated by molecules secreted by the endothelium such as NO, prostacyclin, and prostaglandin D [86], and that under normal conditions the coagulation cascade is self-regulated. Furthermore, molecules secreted by platelets and involved in further platelet activation, such as platelet activating factor, thrombin, and tumor necrosis factor-α (TNF-α), trigger the production of NO by the endothelial monolayer resulting in a negative feedback system which deactivates platelets [87,88,89]. The PAS results correlated with the number of platelets deposited in the scaffolds. There is a significantly lower number of platelets adhered to cell seeded TC scaffolds because of the development of a confluent monolayer. Even though HUVECs formed a monolayer on the TC samples, there were some material fibers exposed for this group likely promoted platelet adhesion. In the nontreated scaffolds, the cells did not cover the entire scaffold, leading to large areas where the material fibers were exposed and the rapid deposition of platelets. According to our findings, there is an inverse relationship between endothelial cell coverage and platelet deposition on the surface. The platelet counts are higher in the scaffolds where the cells are not confluent, and the material is uncovered. Previous studies have shown that endothelial progenitor cells such and hCB-ECs are able to inhibit the attachment and activation of platelets in vitro [90,91]. Moreover, our results are also similar to the study by Brown et al. (2010) who demonstrated that hCB-ECs have superior adhesion and proliferation to vessel-derived cells such as human aortic endothelial cells, and as such, were able to reduce the platelet adhesion and prevent thrombosis in a vein graft [47]. Future studies in our laboratory will utilize immunocytochemistry to further quantify the expression of activated platelet specific markers (e.g., CD62) as well as investigate the effects of shear induced platelet activation using a physiologically realistic pulsatile flow experimental setup.

It should be noted that we did not quantify any of our outcomes on electrospun constructs thermoformed and treated with collagen IV or fibronectin individually. The choice of studying our outcomes on thermoformed and electrospun constructs simultaneously coated with both collagen IV and fibronectin was made after observing the data in Figure S1, which demonstrates that this coating had the highest trend of cell count on coverslips. Therefore, a limitation of our study is that we do not know if the individual coating of collagen IV or fibronectin following thermoforming would have resulted in similar, better, or worse coverage as seen in Figure S2. Another limitation of our study is that all cell experiments were completed under static conditions. Although in vitro endothelialization studies of electrospun scaffolds are primarily performed under static conditions [44,92,93,94], we recognize the importance of evaluating endothelial cell function under physiological shear stress, an important mechanical stimulus controlling cell response [95]. For example, one of the most important functions of endothelial cells in the vasculature is the production of NO, a potent vasodilator and anti-inflammatory mediator [96]. It is reported that shear stress stimulates eNOS production, eNOS phosphorylation, and NO synthesis [97,98,99,100,101]. Therefore, to be able to make further conclusions about the preservation of endothelial cell phenotype of hCB-EC seeded in thermoformed and coated scaffolds, future studies will focus on elucidating growth and functionality under physiological shear stress. Another limitation of our study is that all scaffolds reported were flat sheets and not tubular scaffolds. One of our ongoing studies is to assess hCB-EC cell growth and function in tubular scaffolds, both in static and flow conditions. Future pulsatile flow experiments will be performed in a parallel plate flow chamber for planar scaffolds and in custom tubular bioreactors.

## 5. Conclusions

hCB-ECs cultured on scaffolds composed of polycaprolactone, gelatin, and fibrinogen that are thermoformed and coated with collagen IV and fibronectin are attractive for endothelial cell attachment and cell growth. These cells have the capability to proliferate and form a stable monolayer on the surface of the planar scaffold. Upon monolayer formation, hCB-ECs can produce eNOS, respond to the addition of IL-1β through the upregulation of VCAM-1 and ICAM-1, and reduce platelet deposition and activation rate. Our hCB-ECs have superior cell proliferation but lower inflammatory response when compared to HUVECs in electrospun scaffolds.

The long-term goal of our team is to generate a TEVG that supports endothelial cell growth, is biomechanically matched to a target artery, and remains functional and durable post-implantation for artery replacement. With the present work, we demonstrate that thermoforming and coating on polycaprolactone/gelatin/fibrinogen scaffolds enhances hCB-EC growth and improves maintenance of their EC phenotype in vitro. Future directions will include the development and assessment of layered scaffolds as well as in vivo studies using an aortic implantation to functionally assess our TEVG.

We believe this work provides important findings towards the endothelialization of electrospun scaffolds, and thus will have an impact on the field of tissue engineering and regenerative medicine. We demonstrated that hybrid electrospun scaffolds that were surface modified by the combined treatments of thermoforming and coating and were subsequently seeded with endothelial cells derived from cord blood show promise for vascular tissue engineering.

## Supplementary Materials

The following are available online at http://www.mdpi.com/2077-0383/8/2/185/s1, Table S1. Detailed information on antibodies, Figure S1. Cell number of hCB-ECs 3 days post seeding on glass micro-coverslips coated with collagen type I (ColI), collagen type IV (ColIV), fibronectin (Fib), or a 1:1 mixture of ColI and ColIV (ColI/ColIV), ColI and Fib (ColI/Fib), or ColIV and Fib (ColIV/Fib) in 96-well plates, Figure S2. Maximum intensity projection multiphoton images of hCB-ECs or HUVECs cultured in nontreated, thermoformed, coated, or thermoformed coated scaffolds with collagen IV and fibronectin after 7 days of culture (green—scaffolds; blue—nuclei; red—F-actin), Figure S3. MTS assay calibration curves for hCB-ECs and HUVEC (*n* = 6).

## References

[1] Roger V.L., Go A.S., Lloyd-Jones D.M., Benjamin E.J., Berry J.D., Borden W.B., Bravata D.M., Dai S., Ford E.S., Fox C.S. (2012). Heart disease and stroke statistics—2012 update a report from the American heart association. Circulation.

[2] Mozaffarian D., Benjamin E.J., Go A.S., Arnett D.K., Blaha M.J., Cushman M., Das S.R., de Ferranti S., Després J.-P., Fullerton H.J. (2016). Executive summary: Heart disease and stroke statistics—2016 update a report from the American heart association. Circulation.

[3] Sell S.A., McClure M.J., Garg K., Wolfe P.S., Bowlin G.L. (2009). Electrospinning of collagen/biopolymers for regenerative medicine and cardiovascular tissue engineering. Adv. Drug Deliv. Rev..

[4] Go A.S., Mozaffarian D., Roger V.L., Benjamin E.J., Berry J.D., Blaha M.J., Dai S., Ford E.S., Fox C.S., Franco S. (2014). Heart disease and stroke statistics—2014 update: A report from the American Heart Association. Circulation.

[5] McClure M.J., Sell S.A., Simpson D.G., Walpoth B.H., Bowlin G.L. (2010). A three-layered electrospun matrix to mimic native arterial architecture using polycaprolactone, elastin, and collagen: A preliminary study. Acta Biomater..

[6] McClure M., Wolfe P., Rodriguez I., Bowlin G. (2011). Bioengineered vascular grafts: Improving vascular tissue engineering through scaffold design. J. Drug Deliv. Sci. Technol..

[7] Byrom M.J., Ng M.K., Bannon P.G. (2013). Biomechanics and biocompatibility of the perfect conduit—Can we build one?. Ann. Cardiothorac. Surg..

[8] L’Heureux N., Dusserre N., Marini A., Garrido S., de la Fuente L., McAllister T. (2007). Technology insight: The evolution of tissue-engineered vascular grafts—From research to clinical practice. Nat. Clin. Pract. Cardiovasc. Med..

[9] Tan A., Gundogan B., Farhatnia Y., Nayyer L., Mahdibeiraghdar S., Rajadas J., De Coppi P., Davies A.H., Seifalian A.M. (2015). Tissue engineering vascular grafts a fortiori: Looking back and going forward. Expert Opin. Biol. Ther..

[10] Hasan A., Memic A., Annabi N., Hossain M., Paul A., Dokmeci M.R., Dehghani F., Khademhosseini A. (2014). Electrospun scaffolds for tissue engineering of vascular grafts. Acta Biomater..

[11] Ravi S., Chaikof E.L. (2010). Biomaterials for vascular tissue engineering. Regen. Med..

[12] Dhandayuthapani B., Yoshida Y., Maekawa T., Kumar D.S. (2011). Polymeric scaffolds in tissue engineering application: A review. Int. J. Polym. Sci..

[13] Lannutti J., Reneker D., Ma T., Tomasko D., Farson D. (2007). Electrospinning for tissue engineering scaffolds. Mater. Sci. Eng. C.

[14] Rim N.G., Shin C.S., Shin H. (2013). Current approaches to electrospun nanofibers for tissue engineering. Biomed. Mater..

[15] Nagiah N., Johnson R., Anderson R., Elliott W., Tan W. (2015). Highly compliant vascular grafts with gelatin-sheathed coaxially structured nanofibers. Langmuir.

[16] Fukunishi T., Best C.A., Sugiura T., Shoji T., Yi T., Udelsman B., Ohst D., Ong C.S., Zhang H., Shinoka T. (2016). Tissue-engineered small diameter arterial vascular grafts from cell-free nanofiber pcl/chitosan scaffolds in a sheep model. PLoS ONE.

[17] Pan Y., Zhou X., Wei Y., Zhang Q., Wang T., Zhu M., Li W., Huang R., Liu R., Chen J. (2017). Small-diameter hybrid vascular grafts composed of polycaprolactone and polydioxanone fibers. Sci. Rep..

[18] Tamimi E., Ardila D., Haskett D., Doetschman T., Slepian M.J., Kellar R.S., Geest J.V. (2016). Biomechanical comparison of glutaraldehyde-crosslinked gelatin fibrinogen electrospun scaffolds to porcine coronary arteries. J. Biomech. Eng..

[19] Melchiorri A., Hibino N., Yi T., Lee Y., Sugiura T., Tara S., Shinoka T., Breuer C., Fisher J. (2015). Contrasting biofunctionalization strategies for the enhanced endothelialization of biodegradable vascular grafts. Biomacromolecules.

[20] Waller B.F., Orr C.M., Slack J.D., Pinkerton C.A., Van Tassel J., Peters T. (1992). Anatomy, histology, and pathology of coronary arteries: A review relevant to new interventional and imaging techniques—Part I. Clin. Cardiol..

[21] Michiels C. (2003). Endothelial cell functions. J. Cell. Physiol..

[22] Deanfield J.E., Halcox J.P., Rabelink T.J. (2007). Endothelial function and dysfunction testing and clinical relevance. Circulation.

[23] Zhang H., Tao Y., Ren S., Liu H., Zhou H., Hu J., Tang Y., Zhang B., Chen H. (2017). Isolation and characterization of human umbilical cord-derived endothelial colony-forming cells. Exp. Ther. Med..

[24] Weber B., Zeisberger S.M., Hoerstrup S.P. (2014). Umbilical cord blood-derived endothelial progenitor cells for cardiovascular tissue engineering. Perinatal Stem Cells.

[25] Stroncek J.D., Grant B.S., Brown M.A., Povsic T.J., Truskey G.A., Reichert W.M. (2009). Comparison of endothelial cell phenotypic markers of late-outgrowth endothelial progenitor cells isolated from patients with coronary artery disease and healthy volunteers. Tissue Eng. Part A.

[26] Ingram D.A., Mead L.E., Tanaka H., Meade V., Fenoglio A., Mortell K., Pollok K., Ferkowicz M.J., Gilley D., Yoder M.C. (2004). Identification of a novel hierarchy of endothelial progenitor cells using human peripheral and umbilical cord blood. Blood.

[27] Cai H., Gehrig P., Scott T.M., Zimmermann R., Schlapbach R., Zisch A.H. (2006). MnSOD marks cord blood late outgrowth endothelial cells and accompanies robust resistance to oxidative stress. Biochem. Biophys. Res. Commun..

[28] Bompais H., Chagraoui J., Canron X., Crisan M., Liu X.H., Anjo A., Tolla-Le Port C., Leboeuf M., Charbord P., Bikfalvi A. (2004). Human endothelial cells derived from circulating progenitors display specific functional properties compared with mature vessel wall endothelial cells. Blood.

[29] Schmidt D., Breymann C., Weber A., Guenter C.I., Neuenschwander S., Zund G., Turina M., Hoerstrup S.P. (2004). Umbilical cord blood derived endothelial progenitor cells for tissue engineering of vascular grafts. Ann. Thorac. Surg..

[30] Brown M.A., Wallace C.S., Angelos M., Truskey G.A. (2009). Characterization of umbilical cord blood-derived late outgrowth endothelial progenitor cells exposed to laminar shear stress. Tissue Eng. Part A.

[31] Brown M.E., Rondon E., Rajesh D., Mack A., Lewis R., Feng X., Zitur L.J., Learish R.D., Nuwaysir E.F. (2010). Derivation of induced pluripotent stem cells from human peripheral blood T lymphocytes. PLoS ONE.

[32] Jung Y., Ji H., Chen Z., Fai Chan H., Atchison L., Klitzman B., Truskey G., Leong K.W. (2015). Scaffold-free, human mesenchymal stem cell-based tissue engineered blood vessels. Sci. Rep..

[33] Javed M.J., Mead L.E., Prater D., Bessler W.K., Foster D., Case J., Goebel W.S., Yoder M.C., Haneline L.S., Ingram D.A. (2008). Endothelial colony forming cells and mesenchymal stem cells are enriched at different gestational ages in human umbilical cord blood. Pediatr. Res..

[34] Balasubramanian P., Prabhakaran M.P., Kai D., Ramakrishna S. (2013). Human cardiomyocyte interaction with electrospun fibrinogen/gelatin nanofibers for myocardial regeneration. J. Biomater. Sci. Polym. Ed..

[35] Ardila D.C., Tamimi E., Danford F.L., Haskett D.G., Kellar R.S., Doetschman T., Vande Geest J.P. (2015). TGFβ2 differentially modulates smooth muscle cell proliferation and migration in electrospun gelatin-fibrinogen constructs. Biomaterials.

[36] Koynova R., Antonova B., Sezanova B., Tenchov B.J.T.A. (2018). Beneficial effect of sequential chemotherapy treatments of lung cancer patients revealed by calorimetric monitoring of blood plasma proteome denaturation. Thermochim. Acta.

[37] Biscarat J., Charmette C., Sanchez J., Pochat-Bohatier C. (2015). Preparation of dense gelatin membranes by combining temperature induced gelation and dry-casting. J. Membr. Sci..

[38] Ashton J.H., Mertz J.A., Harper J.L., Slepian M.J., Mills J.L., McGrath D.V., Vande Geest J.P. (2011). Polymeric endoaortic paving: Mechanical, thermoforming, and degradation properties of polycaprolactone/polyurethane blends for cardiovascular applications. Acta Biomater..

[39] Sasidharan A., David A., Gohil A., Gupta A.K. (2015). Simple device to determine the pressure applied by pressure clips for the treatment of earlobe keloids. Indian J. Plast. Surg..

[40] Williams M.J., Utzinger U., Barkmeier-Kraemer J.M., Vande Geest J.P. (2014). Differences in the microstructure and biomechanical properties of the recurrent laryngeal nerve as a function of age and location. J. Biomech. Eng..

[41] Haskett D., Azhar M., Utzinger U., Vande Geest J.P. (2013). Progressive alterations in microstructural organization and biomechanical response in the ApoE mouse model of aneurysm. Biomatter.

[42] Jesty J., Bluestein D. (1999). Acetylated prothrombin as a substrate in the measurement of the procoagulant activity of platelets: Elimination of the feedback activation of platelets by thrombin. Anal. Biochem..

[43] Merkle V.M., Martin D., Hutchinson M., Tran P.L., Behrens A., Hossainy S., Sheriff J., Bluestein D., Wu X., Slepian M.J. (2015). Hemocompatibility of poly (vinyl alcohol)–gelatin core–shell electrospun nanofibers: A scaffold for modulating platelet deposition and activation. ACS Appl. Mater. Interfaces.

[44] Merkle V.M., Tran P.L., Hutchinson M., Ammann K.R., DeCook K., Wu X., Slepian M.J. (2015). Core–shell PVA/gelatin electrospun nanofibers promote human umbilical vein endothelial cell and smooth muscle cell proliferation and migration. Acta Biomater..

[45] Kuwahara M., Sugimoto M., Tsuji S., Matsui H., Mizuno T., Miyata S., Yoshioka A. (2002). Platelet shape changes and adhesion under high shear flow. Arterioscler. Thromb. Vasc. Biol..

[46] Stavropoulos-Giokas C., Charron D., Navarrete C. (2014). Cord Blood Stem Cells Medicine.

[47] Brown M.A., Zhang L., Levering V.W., Wu J.-H., Satterwhite L.L., Brian L., Freedman N.J., Truskey G.A. (2010). Human umbilical cord blood–derived endothelial cells reendothelialize vein grafts and prevent thrombosis. Arterioscler. Thromb. Vasc. Biol..

[48] Zhang L., Yang R., Han Z.C. (2006). Transplantation of umbilical cord blood-derived endothelial progenitor cells: A promising method of therapeutic revascularisation. Eur. J. Haematol..

[49] Chow T., Mueller S., Rogers I.M., El-Badri N. (2017). Advances in umbilical cord blood therapy: Hematopoietic stem cell transplantation and beyond. Advances in Stem Cell Therapy.

[50] Burrows M.C., Zamarion V.M., Filippin-Monteiro F.B., Schuck D.C., Toma H.E., Campa A., Garcia C.R., Catalani L.H. (2012). Hybrid scaffolds built from pet and collagen as a model for vascular graft architecture. Macromol. Biosci..

[51] Ma Z., Kotaki M., Yong T., He W., Ramakrishna S. (2005). Surface engineering of electrospun polyethylene terephthalate (PET) nanofibers towards development of a new material for blood vessel engineering. Biomaterials.

[52] Cassady A.I., Hidzir N.M., Grøndahl L. (2014). Enhancing expanded poly (tetrafluoroethylene) (ePTFE) for biomaterials applications. J. Appl. Polym. Sci..

[53] Takagi H., Goto S.N., Matsui M., Manabe H., Umemoto T. (2010). A contemporary meta-analysis of Dacron versus polytetrafluoroethylene grafts for femoropopliteal bypass grafting. J. Vasc. Surg..

[54] Jeschke M.G., Hermanutz V., Wolf S.E., Köveker G.B. (1999). Polyurethane vascular prostheses decreases neointimal formation compared with expanded polytetrafluoroethylene. J. Vasc. Surg..

[55] MüLLER-HüLSBECK S., Walluscheck K.P., Priebe M., Grimm J., Cremer J., Heller M. (2002). Experience on endothelial cell adhesion on vascular stents and stent-grafts: First in vitro results. Invest. Radiol..

[56] Ren X., Feng Y., Guo J., Wang H., Li Q., Yang J., Hao X., Lv J., Ma N., Li W. (2015). Surface modification and endothelialization of biomaterials as potential scaffolds for vascular tissue engineering applications. Chem. Soc. Rev..

[57] Li Q., Wang Z., Zhang S., Zheng W., Zhao Q., Zhang J., Wang L., Wang S., Kong D. (2013). Functionalization of the surface of electrospun poly (epsilon-caprolactone) mats using zwitterionic poly (carboxybetaine methacrylate) and cell-specific peptide for endothelial progenitor cells capture. Mater. Sci. Eng. C.

[58] Xiong G.M., Yuan S., Tan C.K., Wang J.K., Liu Y., Tan T.T.Y., Tan N.S., Choong C. (2014). Endothelial cell thrombogenicity is reduced by ATRP-mediated grafting of gelatin onto PCL surfaces. J. Mater. Chem. B.

[59] Choi W.S., Joung Y.K., Lee Y., Bae J.W., Park H.K., Park Y.H., Park J.-C., Park K.D. (2016). Enhanced patency and endothelialization of small-caliber vascular grafts fabricated by coimmobilization of heparin and cell-adhesive peptides. ACS Appl. Mater. Interfaces.

[60] Huang Y., Onyeri S., Siewe M., Moshfeghian A., Madihally S.V. (2005). In vitro characterization of chitosan–gelatin scaffolds for tissue engineering. Biomaterials.

[61] Wu S.-C., Chang W.-H., Dong G.-C., Chen K.-Y., Chen Y.-S., Yao C.-H. (2011). Cell adhesion and proliferation enhancement by gelatin nanofiber scaffolds. J. Bioact. Compatible Polym..

[62] D’Souza S.E., Ginsberg M.H., Plow E.F. (1991). Arginyl-glycyl-aspartic acid (RGD): A cell adhesion motif. Trends Biochem. Sci..

[63] Ruoslahti E., Pierschbacher M.D. (1986). Arg-gly-asp: A versatile cell recognition signal. Cell.

[64] Widhe M., Shalaly N.D. (2016). A fibronectin mimetic motif improves integrin mediated cell biding to recombinant spider silk matrices. Biomaterials.

[65] Pierce B.F., Pittermann E., Ma N., Gebauer T., Neffe A.T., Hölscher M., Jung F., Lendlein A. (2012). Viability of human mesenchymal stem cells seeded on crosslinked entropy-elastic gelatin-based hydrogels. Macromol. Biosci..

[66] Underwood P.A., Bennett F.A., Kirkpatrick A., Bean P.A., Moss B.A. (1995). Evidence for the location of a binding sequence for the α2β1 integrin of endothelial cells, in the β1 subunit of laminin. Biochem. J..

[67] Saotome T., Hayashi H., Tanaka R., Kinugasa A., Uesugi S., Tatematsu K.-I., Sezutsu H., Kuwabara N., Asakura T. (2015). Introduction of VEGF or RGD sequences improves revascularization properties of bombyx mori silk fibroin produced by transgenic silkworm. J. Mater. Chem. B.

[68] Ingber D.E. (1990). Fibronectin controls capillary endothelial cell growth by modulating cell shape. Proc. Natl. Acad. Sci. USA.

[69] Bahou W.F., Potter C.L., Mirza H. (1994). The VLA-2 (alpha 2 beta 1) I domain functions as a ligand-specific recognition sequence for endothelial cell attachment and spreading: Molecular and functional characterization. Blood.

[70] Chen M., Patra P.K., Warner S.B., Bhowmick S. (2007). Role of fiber diameter in adhesion and proliferation of NIH 3T3 fibroblast on electrospun polycaprolactone scaffolds. Tissue Eng..

[71] Whited B.M., Rylander M.N. (2014). The influence of electrospun scaffold topography on endothelial cell morphology, alignment, and adhesion in response to fluid flow. Biotechnol. Bioeng..

[72] Adams W.J., Zhang Y., Cloutier J., Kuchimanchi P., Newton G., Sehrawat S., Aird W.C., Mayadas T.N., Luscinskas F.W., García-Cardeña G. (2013). Functional vascular endothelium derived from human induced pluripotent stem cells. Stem Cell Rep..

[73] Van Rijssel J., Timmerman I., Van Alphen F.P., Hoogenboezem M., Korchynskyi O., Geerts D., Geissler J., Reedquist K.A., Niessen H.W., Van Buul J.D. (2013). The Rho-GEF Trio regulates a novel pro-inflammatory pathway through the transcription factor Ets2. Biol. Open.

[74] Takahashi M., Ikeda U., Masuyama J.-I., Kitagawa S.-I., Kasahara T., Shimpo M., Kano S., Shimada K. (1996). Monocyte-endothelial cell interaction induces expression of adhesion molecules on human umbilical cord endothelial cells. Cardiovasc. Res..

[75] Blake G.J., Ridker P.M. (2001). Novel clinical markers of vascular wall inflammation. Circul. Res..

[76] Qin L., Huang Q., Zhang H., Liu R., Tellides G., Min W., Yu L. (2014). Socs1 prevents graft arteriosclerosis by preserving endothelial cell function. J. Am. Coll. Cardiol..

[77] Zou Y., Hu Y., Mayr M., Dietrich H., Wick G., Xu Q. (2000). Reduced neointima hyperplasia of vein bypass grafts in intercellular adhesion molecule-1–deficient mice. Circul. Res..

[78] Yuan Y., Stewart D.J., Courtman D.W. (2016). The regulation of endothelial nitric oxide synthase by extracellular matrix in human late outgrowth endothelial progenitor cells. Front. Bioeng. Biotechnol. Conference Abstract: 10th World Biomaterials Congress, Montréal, Canada, 17–22 May, 2016.

[79] Viji R., Kumar V.S., Kiran M., Sudhakaran P. (2009). Modulation of endothelial nitric oxide synthase by fibronectin. Mol. Cell. Biochem..

[80] Matsushita H., Chang E., Glassford A.J., Cooke J.P., Chiu C.-P., Tsao P.S. (2001). eNOS activity is reduced in senescent human endothelial cells preservation by hTERT immortalization. Circul. Res..

[81] Tran J., Magenau A., Rodriguez M., Rentero C., Royo T., Enrich C., Thomas S.R., Grewal T., Gaus K. (2016). Activation of endothelial nitric oxide (eNOS) occurs through different membrane domains in endothelial cells. PLoS ONE.

[82] Ruan T., Bharath L., Mueller R., Goodrich R., Graham T., Symons J.D. (2015). Shear-induced extracellular regulated kinase signaling to eNOS is increased when autophagy is compromised in endothelial cells. FASEB J..

[83] Li Y., Zheng J., Bird I.M., Magness R.R. (2005). Effects of pulsatile shear stress on signaling mechanisms controlling nitric oxide production, endothelial nitric oxide synthase phosphorylation, and expression in ovine fetoplacental artery endothelial cells. Endothelium.

[84] Yang B., Rizzo V. (2013). Shear stress activates eNOS at the endothelial apical surface through β1 containing integrins and caveolae. Cell. Mol. Bioeng..

[85] Do Kang S., Carlon T.A., Jantzen A.E., Lin F.-H., Ley M.M., Allen J.D., Stabler T.V., Haley N.R., Truskey G.A., Achneck H.E. (2013). Isolation of functional human endothelial cells from small volumes of umbilical cord blood. Ann. Biomed. Eng..

[86] Broos K., Feys H.B., De Meyer S.F., Vanhoorelbeke K., Deckmyn H. (2011). Platelets at work in primary hemostasis. Blood Rev..

[87] Szabo C. (1995). Alterations in nitric oxide production in various forms of circulatory shock. New Horiz..

[88] Van Hinsbergh V.W. (2012). Endothelium—Role in regulation of coagulation and inflammation. Semin. Immunopathol..

[89] Cronenwett J.L., Johnston K.W. (2014). Rutherford’s Vascular Surgery.

[90] Abou-Saleh H., Yacoub D., Théorêt J.-F., Gillis M.-A., Neagoe P.-E., Labarthe B., Théroux P., Sirois M.G., Tabrizian M., Thorin E. (2009). Endothelial progenitor cells bind and inhibit platelet function and thrombus formation. Circulation.

[91] Shirota T., He H., Yasui H., Matsuda T. (2003). Human endothelial progenitor cell-seeded hybrid graft: Proliferative and antithrombogenic potentials in vitro and fabrication processing. Tissue Eng..

[92] He W., Yong T., Teo W.E., Ma Z., Ramakrishna S. (2005). Fabrication and endothelialization of collagen-blended biodegradable polymer nanofibers: Potential vascular graft for blood vessel tissue engineering. Tissue Eng..

[93] Lee B., Shafiq M., Jung Y., Park J.-C., Kim S.H. (2016). Characterization and preparation of bio-tubular scaffolds for fabricating artificial vascular grafts by combining electrospinning and a co-culture system. Macromol. Res..

[94] Zhou W., Feng Y., Yang J., Fan J., Lv J., Zhang L., Guo J., Ren X., Zhang W. (2015). Electrospun scaffolds of silk fibroin and poly (lactide-co-glycolide) for endothelial cell growth. J. Mater. Sci. Mater. Med..

[95] Li Y.-S.J., Haga J.H., Chien S. (2005). Molecular basis of the effects of shear stress on vascular endothelial cells. J. Biomech..

[96] Sessa W.C. (2004). Enos at a glance. J. Cell. Sci..

[97] Kolluru G.K., Sinha S., Majumder S., Muley A., Siamwala J.H., Gupta R., Chatterjee S. (2010). Shear stress promotes nitric oxide production in endothelial cells by sub-cellular delocalization of eNOS: A basis for shear stress mediated angiogenesis. Nitric Oxide.

[98] Dimmeler S., Fleming I., Fisslthaler B., Hermann C., Busse R., Zeiher A.M. (1999). Activation of nitric oxide synthase in endothelial cells by Akt-dependent phosphorylation. Nature.

[99] Uematsu M., Ohara Y., Navas J.P., Nishida K., Murphy T., Alexander R.W., Nerem R.M., Harrison D.G. (1995). Regulation of endothelial cell nitric oxide synthase mRNA expression by shear stress. Am. J. Physiol..

[100] Buga G.M., Gold M.E., Fukuto J.M., Ignarro L.J. (1991). Shear stress-induced release of nitric oxide from endothelial cells grown on beads. Hypertension.

[101] Hsieh H.-J., Liu C.-A., Huang B., Tseng A.H., Wang D.L. (2014). Shear-induced endothelial mechanotransduction: The interplay between reactive oxygen species (ROS) and nitric oxide (NO) and the pathophysiological implications. J. Biomed. Sci..

