# Supplementary material for: Surface Modification of Electrospun Scaffolds for Endothelialization of Tissue-Engineered Vascular Grafts Using Human Cord Blood-Derived Endothelial Cells
Source: J Clin Med. 2019 Feb 4;8(2):185. doi: 10.3390/jcm8020185 (PMC6416564; doi:10.3390/jcm8020185)
Supplement: Supplementary file 1 [file jcm-08-00185-s001.pdf]

**Table S1.** Detailed information on antibodies.

| Antibody | Company | Catalog Number | Concentration | Application |
|---|---|---|---|---|
| CD31 | BD Biosciences | 555445 | 20 μL/test | Flow cytometry, immunochemistry |
| CD45 | BD Biosciences | 562394 | 5 μL/test | Flow cytometry |
| CD105 | BD Biosciences | 562408 | 5 μL/test | Flow cytometry |
| Intercellular adhesion molecule 1 (ICAM-1) | Abcam | ab2213 | 1/100 | ELISA |
| Vascular cell adhesion molecule 1 (VCAM-1) | Abcam | ab98954 | 1/100 | ELISA |
| Endothelial nitric oxide synthase (eNOS) | Abcam | ab76198 | 1/1000 | ELISA |
| Rabbit anti-mouse secondary antibody | Abcam | ab6728 | 1/1000 | ELISA |

ELISA: Enzyme-linked immunosorbent assay.

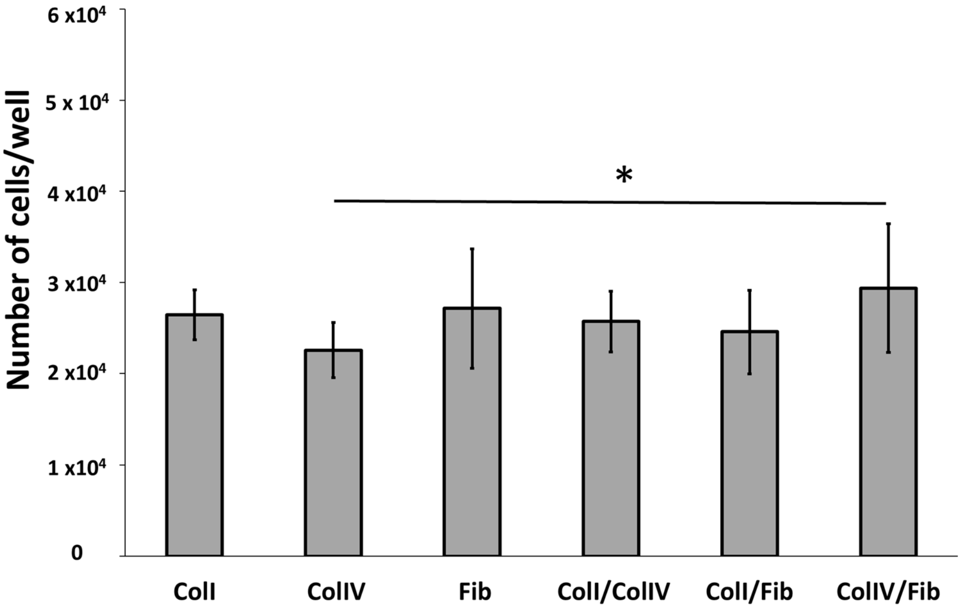


**Figure S1.** Cell number of hCB-ECs 3 days post seeding on coverslips coated with collagen type I (ColI), collagen type IV (ColIV), fibronectin (Fib), or a 1:1 mixture of ColI and ColIV (ColI/ColIV), ColI and Fib (ColI/Fib), or ColIV and Fib (ColIV/Fib) in 96-well plates. Error bars shown are standard deviations (* $p < 0.05$; $n = 6$). The combination of collagen IV and fibronectin promotes hCB-EC growth when compared to collagen IV alone.

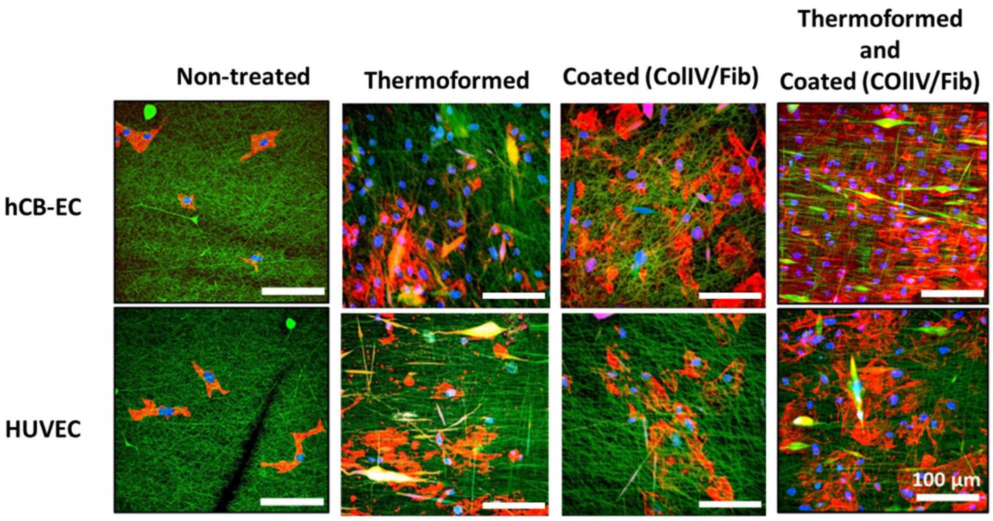


**Figure S2.** Maximum intensity projection multiphoton images of Human cord blood-derived endothelial cells (hCB-ECs) or human umbilical vein endothelial cells (HUVECs) cultured in non-treated, thermoformed, coated with collagen IV and fibronectin, or thermoformed and coated scaffolds after 7 days of culture (green—scaffolds; blue—nuclei; red—F-actin). Scale bar = 100 µm. The thermoformed and coated scaffolds are the most favorable for cell attachment and cell spreading compared to thermoformed alone or coated alone in both cell types.

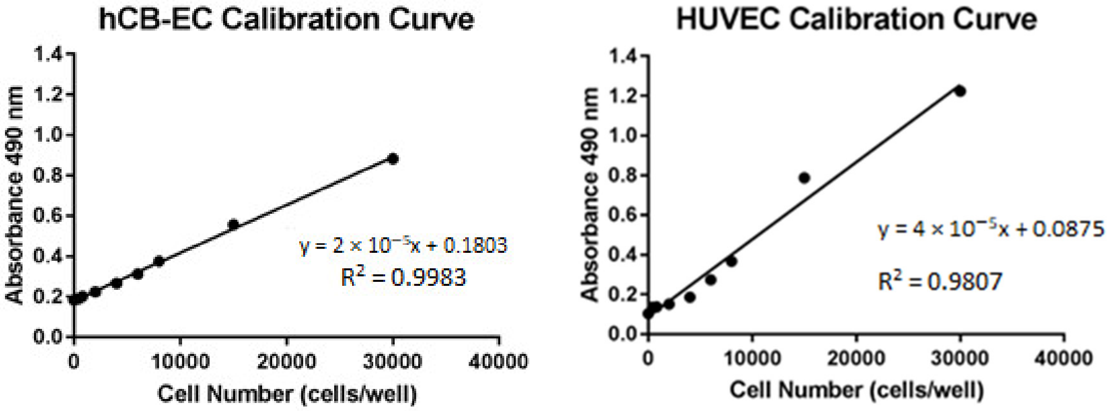


**Figure S3.** MTS assay calibration curves for hCB-EC and HUVEC ($n$ = 6).
